# The Less Expensive Choice: Bacterial Strategies to Achieve Successful and Sustainable Reciprocal Interactions

**DOI:** 10.3389/fmicb.2020.571417

**Published:** 2021-01-20

**Authors:** Enrica Pessione

**Affiliations:** Department of Life Sciences and Systems Biology, Università degli Studi di Torino, Torino, Italy

**Keywords:** economize, sharing, storing, cooperative behaviors, system communication

## Abstract

Bacteria, the first organisms that appeared on Earth, continue to play a central role in ensuring life on the planet, both as biogeochemical agents and as higher organisms’ symbionts. In the last decades, they have been employed both as bioremediation agents for cleaning polluted sites and as bioconversion effectors for obtaining a variety of products from wastes (including eco-friendly plastics and green energies). However, some recent reports suggest that bacterial biodiversity can be negatively affected by the present environmental crisis (global warming, soil desertification, and ocean acidification). This review analyzes the behaviors positively selected by evolution that render bacteria good models of sustainable practices (urgent in these times of climate change and scarcity of resources). Actually, bacteria display a tendency to optimize rather than maximize, to economize energy and building blocks (by using the same molecule for performing multiple functions), and to recycle and share metabolites, and these are winning strategies when dealing with sustainability. Furthermore, their ability to establish successful reciprocal relationships by means of anticipation, collective actions, and cooperation can also constitute an example highlighting how evolutionary selection favors behaviors that can be strategic to contain the present environmental crisis.

## Introduction

The role of microorganisms as actors in environmental sustainability has long been established (Kuhad, 2012). Since the last century, both bacteria and fungi have been employed in bioremediation of polluted sites with particular reference to hydrocarbons and heavy metals (Dash et al., 2013). In the changing environment of the 21st century, deeply marked by environmental challenges such as climate change, resource exhaustion, and demographic pressure, advances in applied research allow exploiting the microbial potential to maintain favorable conditions for life on the Earth. Bacteria can be used in the bioconversion of wastes for (i) generation of renewable energy (microbial fuel cells, methane, biobutanol, bioethanol, and biohydrogen production) (Cho et al., 2020; Mazzoli et al., 2012), (ii) synthesis of bio-based and biodegradable plastic polymers from lignocellulosic wastes (Mazzoli et al., 2014; Abdel-Rahman and Sonomoto, 2016), (iii) production of antibiotic alternatives (Cotter et al., 2013), and (iv) supporting organic agriculture and reducing greenhouse gases (methanotrophic archaea and photosynthetic plankton) (Kuhad, 2012).

However, it has been recently pointed out that microbial communities can suffer from unfavorable life conditions such as those arising from the over-exploitation of soils in conventional intensive agriculture (Kuhad, 2012) and from the growing global warming (Cavicchioli et al., 2019). Actually, the reduction of microbial biodiversity could impact the ability of other species to survive in both terrestrial and aquatic environments, because changes at the microbial community level can affect overall ecosystem biochemical fluxes important in supporting biogeochemical cycles and, ultimately, life on the planet (Cavicchioli et al., 2019). Although bacteria, as extensively reported in the present review, display excellent capabilities to adapt to changing conditions, their potential to face a very high number of environmental challenges can be threatened.

The present review explores a different standpoint on the role of bacteria in sustainability: considering them not as effectors of eco-friendly technological approaches but as models of behaviors that were positively selected by a very long evolutionary history and are still successful. Fast-changing environments, especially for non-symbiotic microorganisms, are the rule in bacterial life, as well as resource exhaustion and demographic pressure. In this scenario, their tendency for optimization rather than maximization, their interconnections, cross-feeding, sharing, and recycling together with their propensity to economize can be seen as valuable examples of successful ways of living, even in scarcity of resources and in rapid environmental changes as those we are currently facing.

Here, I will first try to examine the concept of bacterial intelligence and then I will describe some examples of cheap strategies and cooperative behaviors set up by bacteria at different levels to reach better fitness and more complex degree of organization.

## Bacterial Intelligence and Interactive Connections

Although the term intelligence is generally applied to brain-bearing animals, evidence supports the idea of different forms of intelligence in brainless living organisms such as plants (Trewavas, 2002) and microbes (Hellingwerf, 2005). As reported by Shapiro (2007), bacteria are “small but not stupid”: they can explore their environment and then adapt accordingly to optimize survival and fitness. The more complex are the external conditions, the more sophisticated is the network to cope with. Not only the genetic size (especially richness/abundance in genes encoding proteins involved in signal transduction) but also long-term (transcription and translation control) and short-term (post-translational modifications, proteolysis, allosteric effects) regulations can account for this adaptation abilities.

This intelligence is not an immaterial concept but is based on precise molecular structures and devices (for the role of molecular conformational states in bacterial intelligence, see Westerhoff et al., 2014; for exhaustive review on bacterial intelligence and signal transduction systems in prokaryotes, see Lyon, 2015; Pinto and Mascher, 2016). The module “sense, integrate information and coherently respond,” also called adaptive behavior, is a well-recognized form of intelligence based on the same scheme as neurons, largely spread in bacteria where a sensory receptor, an information processing unit and a motor activity constitute the simplest system sometimes referred as “nanobrain” (Webre et al., 2003). As an example, in *Escherichia coli*, the flagellar motor turns clockwise generating tumbling movements. When a signal molecule (i.e., a nutrient) is sensed by a surface receptor, a phosphorylation transduction cascade is used to trigger counterclockwise rotation of flagellar motor, thus allowing swimming motility toward the nutrient (chemotaxis) (Bourret and Stock, 2002). A protein-based information processing unit constituting a sort of “sensing–integrating–responding” system is the stressosome found in *Bacillus subtilis.* The latter is a micro-organ with higher degree of complexity than the simple motility device finalized to chemiotaxis, able to set differentiated responses to different stressors (Marles-Wright et al., 2008). Additional functional possibilities can be found in prokaryotes such as memory (Wolf et al., 2008), learning (Hoffer et al., 2001), anticipation (Mitchell et al., 2009; Goo et al., 2012), decision making (Adler and Tso, 1974; Ross-Gillespie and Kümmerli, 2014), coordinated movements (Kaiser and Warrick, 2014), and cooperative interactions (Jacob et al., 2004).

**FIGURE 1:**
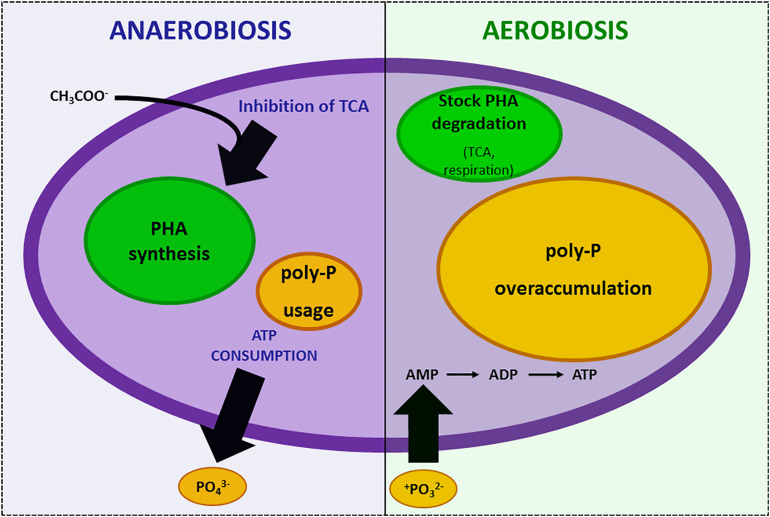
Example of “memory” in strictly aerobic *Acinetobacter* sp. during anaerobiosis–aerobiosis cycles. In anaerobiosis, carbon nutrients cannot be metabolized in the TCA cycle and are accumulated as polyhydroxyalkanoates (PHA) while cells consume all the ATP and phosphorus stocks. This stress condition induces luxury uptake of phosphorus when oxygen availability restores respiration and over-accumulation of phosphate as polyphosphate (poly-P).

The concept *memory–learning–anticipate* strongly underlines that prokaryote reactions take into account not only space (the environment) but also time (Tagkopoulos et al., 2008; Mitchell et al., 2009; Lyon, 2015; Pinto and Mascher, 2016). As an example, saving resources for the future is a possible mechanism elicited by the “awareness” of possible future scarcity. When environmental conditions (e.g., anaerobiosis) inhibit TCA cycle and make ATP synthesis impossible, *Acinetobacter* strains consume all the cellular ATP to survive and store carbon resources as polyhydroxylkanoates (PHA) (Deinema et al., 1980). Meanwhile, they use this steady-state period to activate a “luxury uptake system” of phosphorus that will allow them an “overplus accumulation” of phosphorus into polyphosphates when oxygen is supplied and normal energetic conditions are restored (Deinema et al., 1980). In this second phase, stocked PHA are consumed and ATP synthesis can proceed. Hence, phosphorus is not only used to restore the ATP stock but also intracellularly accumulated as polyphosphate (Deinema et al., 1980). This strategy protects bacteria from further starvation risks (Figure 1). The duration of memory (i.e., ability to retain and store information) varies depending on the situation, from the time necessary for protein modification to long-term (transgenerational) epigenetic modifications (Wolf et al., 2008). Learning (i.e., the ability to assimilate new information) is based on gene/operon autoamplification (Hoffer et al., 2001) and represents the obligate path to be able to respond with anticipatory behavior.

As far as *problem solving–decision making* is concerned, there are several examples highlighting that bacteria can sense the environment and evaluate the costs and benefits, generally making the less expensive choice. In *Pseudomonas aeruginosa*, there are two different siderophores: pyochelin is a low-cost (only six genes involved in its biosynthesis) and low-efficiency system and pyoverdine is a high-cost (14 genes involved) and high-efficiency device. It has been demonstrated that this bacterium normally utilizes the low-cost low-efficiency mechanism using the high-cost high-efficiency system only in extreme iron-limiting conditions (Dumas et al., 2013). Similarly, *E. coli* can “choose” among three different systems to uptake/assimilate ammonia that are hierarchically regulated to prevent ATP waste and futile cycles (van Heeswijk et al., 2013). These two examples underline how evolution has shaped bacterial intelligence to limit energy loss and waste production.

A further degree of complexity concerns the so-called *bacterial social intelligence*, i.e., capability of bacteria to cross-talk and to *act collectively* as observed in population decision making (Goo et al., 2012), movement coordination (swarming) (Kearns, 2010), and cooperation (Tarnita, 2017). These collective behaviors, which include both synergies and conflicts of interest, require coordination and flexibility since the external conditions may rapidly change and the choice between a “faster-and-less-accurate” response (if there is need of rapid but transient coordination) and “more-accurate-but-slower” response (in case of necessity to maintain long-term cooperation) should be clearly established (Ross-Gillespie and Kümmerli, 2014).

**FIGURE 2:**
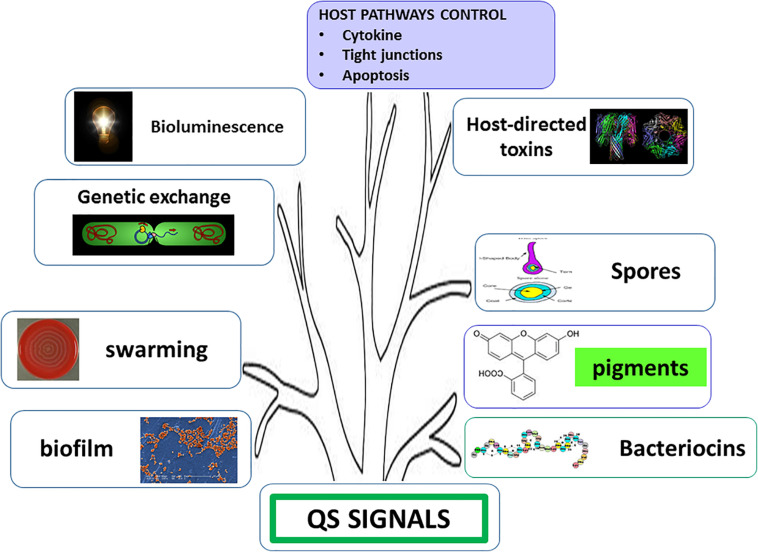
Phenotypic traits under quorum sensing (QS) control. Enhanced interbacterial cooperation (genetic exchange, swarming, and biofilm production) and antagonism (production of pigments and bacteriocins), increased environmental resistance (sporulation), and host cross-talk are induced by QS signals.

The discovery of quorum sensing (QS) (Fuqua et al., 1994; Miller and Bassler, 2001) has shed light on a phenomenon known among humans as “unity is strength.” Collective decision making has been successfully selected by evolution since individual actions have generally low impact and are seldom winning strategies. If a low number of bacterial cells begins to produce toxins or bacteriocins, the most probable event is the defeat. Actually, host immune system or surrounding bacteria sense this and rapidly react accordingly. Briefly, the result is that the attacking cells become victims. If, conversely, the cells begin to produce and secrete weapons when they are in significant number, it is probable that the battle will be won. Once a threshold concentration of diffusible autoinducers (molecules of different chemical structure, for comprehensive reading see Visick and Fuqua, 2005) is reached, revealing that the population number is high, sensing and amplification phenomena occur, thus triggering transcriptional responses resulting in several phenotypic changes such as light or pigment production, bacteriocin/toxin release, competence, sporulation and biofilm formation (Figure 2). Sometimes, the system is so sophisticated that different virulence factors are sequentially produced to obtain a time course effect (Waters and Bassler, 2005). In complex ecosystems, bacteria have to select QS signals from a background noise created by the large number of molecules present in the environment; however, despite these disturbances, feedbacks reveal that this mechanism is able to set up and optimize complex responses as well as to coordinate social behaviors such as collective decision making (Visick and Fuqua, 2005).

A paradigmatic example of collective anticipative behavior is described in *Burkholderia.* When a QS-mediated information is spread in the population, revealing that a threshold biomass is reached and stationary phase is approaching, cells begin to synthesize oxalic acid. This is achieved in conditions of neutral pH (and therefore not as a simple mechanistic response induced by high pH) to preventively buffer alkalization that will occur in stationary phase due to ammonia accumulation following cell lysis and protein/amino acid degradation (Goo et al., 2012). Similarly, a collective decision based on QS and concerning competence occurs in *B. subtilis* where only some cells in the overall population acquire the “competent cell” phenotype (Leisner et al., 2008).

Regarding motility, apart from passive movements especially observed in non-flagellated bacteria (gliding, darting, sliding, floating, and twitching) (Jarrell and McBride, 2008), generally chemotactic behavior is achieved by individual swimming (Bourret and Stock, 2002). Besides this, coordinated collective motility known as swarming is present in several Gram-negative Enterobacteria such as *Proteus* and *Salmonella* (Kim and Surette, 2004; Sturgill and Rather, 2004). Swarming motility is not chemotactic but set up as a means to facilitate access to nutrients and oxygen to all population components, thus preventing intercellular competition (Kaiser and Warrick, 2014). Individual cells align each other (like birds during migration flights) using type IV pili, thus giving origin to a structured expanded movement where the motility apparatus continuously changes position, causing a reverse of direction about every 8 min, probably finalized to avoid collisions. In this mechanism, best studied in *Myxococcus xanthus*, about 40 genes are involved, including a cytoplasmic protein acting as a “pacemaker” (Kaiser and Warrick, 2011). Swarming bacteria are phenotypically different from swimming cells: proteomic analyses revealed that, in *Salmonella*, several proteins are 5- to 20-fold differentially abundant and swarmers displayed higher antibiotic resistance (Kim and Surette, 2004). In *Proteus mirabilis*, the phenotypic changes observed during swarming are controlled by putrescine (the decarboxylation product of ornithine) that act as a signaling system (Sturgill and Rather, 2004).

Discussing cooperative behaviors in bacteria is challenging because cooperation and competition are often intertwined, and the former does not exclude conflicts. In general, cooperation benefits have to outweigh cooperation costs considering that both environmental and time-related factors are crucial in driving eco-evolutionary forces. Therefore, defining bacterial social behavior without a clear ecological context is erroneous (Werner et al., 2014; Tarnita, 2017). According to some authors, synthesizing antagonistic molecules is expensive and decrease biomass production. Therefore, competition or selfish behavior is confined to periods of nutrient abundance (Mora et al., 2013). These authors describe how, in lactic acid bacteria (LAB), the abundance of the carbon substrate has shaped the phenotype from cooperative to selfish. Based on the assumption that respiration is a cooperative behavior allowing sharing of resources in the ecological niche, whereas fermentation is a selfish behavior consuming resources and producing antagonistic compounds (the pH-lowering lactic and acetic acid), the authors speculate on the evolutionary origin of these alternative pathways. Actually, most food-related LAB such as *Streptococcus thermophilus* have lost the capability (present in other pathogenic streptococci best adapted to the animal ecological niche) to synthesize toxins (hemolysins) able to subtract heme from the animal host. For this reason, they cannot have functional respiratory chains (cytochromes contain heme) and are compelled to perform lactic fermentation or mixed-type fermentation (Pessione, 2012). Their adaptation to the milk environment has occurred because, in parallel with the loss of toxin-encoding genes, they have acquired genes for lactose uptake and utilization together with genes involved in pH balancing (Arioli et al., 2010). The overall consequence is that food-related LAB display an antagonistic behavior consuming nutrient resources and producing pH-lowering compounds, while in different conditions, LAB can cooperate with their partners sharing the same ecological niche. Nevertheless, according to other authors, competition increases during scarcity of nutrients and harsh conditions (Escalante and Travisano, 2017). An example is the emergence of GASP cells in *E. coli* during stationary phase. In general, in nutrient scarcity and waste accumulation conditions, wild-type cells stop growing to control population density, provide a slow decay, and guarantee long-term sustainability. However, GASP mutants with selfish behavior, lacking growth inhibitory mechanisms, appear, thus accelerating abrupt death due to overcrowding (ecological collapse) (Vulić and Kolter, 2001). In spite of these opposite considerations, it is well recognized that conflict resolution and adaptive cooperation to maintain phenotypic diversity is a winning road to increase biological complexity during evolution, including major evolutionary transitions toward multicellular organisms (Escalante and Travisano, 2017). Several examples concerning the trend toward cooperation, biofilm development, and maintenance will be examined in subsequent sections.

## Cooperative Strategies: So Advantageous and So Difficult to Achieve

**FIGURE 3:**
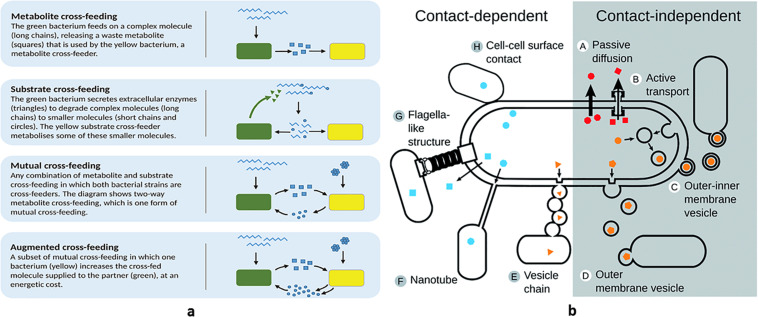
Bacterial syntrophic and interactive behaviors: examples of **(a)** syntrophies and **(b)** metabolite exchange.

Mutual benefit and altruism are the two types of cooperative behavior. Mutual cross-feeding (reciprocal exchange of nutrients) and syntrophic chains (one species utilizes the waste end products of another species supplying its wastes to a third one, and so on, in a vertical temporal sequence and receiving fitness benefits at each step) (Smith et al., 2019) are reciprocally advantageous, supporting optimized exploitation of resources and reducing waste accumulation in a certain ecological niche (Figure 3a). Sulfur-reducing and sulfur-oxidizing bacteria spatially distribute in consortia facilitating metabolite exchanges (Müller and Overmann, 2011). These evolution-driven behaviors from one side can be exploited in the circular economy approach to sustainability (e.g., converting carbon wastes into methane, through a long synthrophic chain that includes acidogenic and acetogenic bacteria), from another they can be seen as examples of sharing and recycling with minimal waste production. In addition to nutritional cooperation, when preceded by gene duplication, gene exchange and recombination is a reciprocally advantageous event as well, supporting all the population with new possibilities to cope with the external environment (capability to utilize new nutrients and antibiotic resistance).

Conjugation offers an emblematic example of intelligent cross-talk finalized to enhance population fitness. Actually, plasmid-bearing donors are not always ready to transfer their DNA to recipients, demonstrating that conjugation is not a simple mechanistic and random event. To convert donors into transfer competent cells, the two partners have to communicate. In the *E. faecalis* model, the recipient secretes peptide pheromones that, once up-taken by the donor cell, induce transcription of the conjugative apparatus by removing a transcriptional repressor (Clewell, 2011). Conversely, in the *B. subtilis* model is the donor that secretes an inhibitory peptide that, once taken up by the recipient, allows the regulatory network to act inducing expression of the gene transfer apparatus (Singh et al., 2013). In both these ways, plasmid is successfully transferred, avoiding random distribution. Furthermore, since transfer-competent cells bear some disadvantages such as increased risk to be attacked by phages at the pilus level (Frost and Koraimann, 2010), higher sensitivity to bile salts (Bidlack and Silverman, 2004), and general stressors (Zahrl et al., 2006), this condition is triggered only when strictly necessary and this is beneficial for both the donor and the recipient. An additional mechanism concerning gene transfer is that in the overall population of potential donors, only a small fraction (0.1–1%) is converted into transfer-competent cells (Wagner et al., 2013). This allows the majority of cells to maintain the plasmid (even for many generations) without paying the costs of its replication and of the expression of the transfer machinery (Koraimann and Wagner, 2014). Whether this subpopulation of transfer-competent cells arises randomly or is affected by environmental/phenotypic factors (e.g., position of the colony in solid media) has to be fully elucidated yet (Reisner et al., 2012).

A more recently discovered mechanism that supports both nutrient sharing and genetic exchanges is the one linked to membrane vesicles. This system also ensures communication between bacterial cells, since, inside the vesicles, different cargo molecules can be transported (nutrients, DNA, and hydrophobic QS peptides) (Toyofuku et al., 2020). Vesicles are present in Gram-negative and Gram-positive bacteria and can originate both in the outer (Manning and Kuehn, 2011) and in the cytoplasmic membrane (Lee et al., 2009). The role of lysogenic phages in membrane vesicle biogenesis has been ascertained and recently reviewed (Pessione, 2020). The system, used to translocate the transported molecules directly inside the target cell by means of membrane fusion, proved to be more efficient than simple metabolite secretion since inside the vesicles the different compounds are protected both from enzymatic degradation and from host factors (for instance, quorum quenching in case of symbiont bacteria) (Figure 3b). Thus, membrane vesicles proved to be useful tools to avoid loss of important and costly molecules.

Sometimes, however, mutual benefits can shift into altruistic behaviors that can damage the effectors and, over long time, the overall population. This is the case of the so-called shared good (or public goods) that especially in liquid habitats can easily diffuse and reach a relative distance from the producer. In *Pseudomonas*, it has been demonstrated that the production of the siderophore pyoverdine can enhance fitness, facilitating iron uptake and utilization, especially in iron-limiting conditions. However, these molecules are not cell-bound but released into the external environment thus becoming accessible for surrounding bacteria (West and Buckling, 2003). This sharing could be advantageous if all the cells contribute to the public good secretion. On the contrary, some mutant cells (called cheaters and consisting in about 9% of the population) emerge that benefit of the secreted siderophores without contributing to their production (Butaité et al., 2017). Among these mutants, some have lost their pyoverdine-synthesis cluster (structural non-producers) whereas others display a complete but inactive locus, producing only residual amounts of the siderophore (silent non-producers). Because of the lower effort in pyoverdine biosynthesis, cheaters have better opportunity to use energy for duplication, thus becoming more and more abundant in the overall population. When this occurs in liquid cultures, the original siderophore producers go extinct and the gene for siderophore biosynthesis is soon lost, rendering the community unable to survive during iron scarcity (Griffin et al., 2004). Despite this so-called “tragedy of the commons,” possible escape lanes for saving the altruistic and therefore the overall population have been reported: (i) presence of low-affinity iron receptors in the cheaters not allowing them to outcompete the pyoverdine producers and (ii) appearance of producers secreting pyoverdines that repress rather than promote the growth of cheaters (Butaité et al., 2017). These resistance mechanisms toward cheaters finally support the cooperative behavior favoring maintenance of biodiversity in the bacterial community.

Different strategies to control cheaters and to specifically direct nutrients toward selected partners have been described, all implicating spatial structuring that limits public good diffusion (Lion and Baalen, 2008). Several examples underline the importance of the close proximity of bacterial cells to achieve the best performance: hydrogen donors and autotrophic methanogenic Archea organize in flocks (Boetius et al., 2000) and a peculiar physical connection through pili that provides direct electron transfer between bacteria (microbial nanowires) also exists (Summers et al., 2010) (Figure 3b). However, the best solution for limiting loss of shared good and for facilitating time-course cooperation is attachment to (biotic and abiotic) solid surfaces as it occurs in biofilms (Werner et al., 2014). This strategy, discussed in a succeeding section, also prevents wasting of precious and costly molecules.

Actually, a different scenario is observed in solid environments, suggesting that the sessile way of life is a fundamental step of evolution that can partly control the cheater damage, reduce energetic costs, and favor cooperative behaviors. Actually, in solid media, surrounding cells are “relatives” (i.e., offspring of the parent cell); hence, producers share the public good with genetically related cells, rendering the behavior (resulting in indirect fitness effects = kin selection) not altruistic but mutualistic (Hamilton, 1964). A further more complex mechanism to isolate cheaters is the cell spatial distribution on solid surfaces, where an assembling of cooperative cells minimizes parasitic behaviors (Kovács, 2014). It has been reported that EPS-producing *B. subtilis* (i.e., those able to generate a biofilm) are advantageously selected over EPS non-producers only if high spatial segregation occurs (thus allowing EPS to be available only to cooperative cells), whereas in low-assortment conditions, cheaters can be favored due to public good availability and lower energetic investment in EPS production. Furthermore, in this model, optimization rather than maximization of EPS production is observed, suggesting that the fitness costs required by the biosynthetic effort is high (Van Gestel et al., 2014). An important factor favoring segregation is the high amount of nutrients: in nutritionally poor media, cross-feeding becomes necessary, thus facilitating low assortment. Even in this case, however, exclusion of non-producers can also occur in mixed communities if a preferential cross-feeding between two cooperators is established (Kovács, 2014). All these mechanisms ensure that good-sharing attitude will not be lost, without the necessity to neutralize cheaters by secreting antimicrobial compounds.

## Bacterial Biofilms: A Model of “High Freedom Degree” Community?

Besides being considered for long time as unicellular organisms, bacteria revealed a complex social organization based on partial differentiation of cell types and exchanging a huge number of data through a complex network of both hydrophobic and water-soluble molecular signals (Shapiro, 1998). This community, called biofilm, has the merit to have depicted in an unambiguous way that cooperative behavior is as important as competition in the evolution of microbial living systems (Crespi, 2001). Actually, biofilm is a successful systemic structure anchored to biotic or abiotic surfaces (O’Toole et al., 2000) that allows nutrient sharing (Costerton et al., 1995), favors genetic exchanges among single cells (Merod and Wuertz, 2014), and protects bacteria from exogenous stressors like animal immune systems (Clutterbuck et al., 2007) and antibiotics (Olsen, 2015). Biofilm lifestyle constitutes, after all, the preferred way of living of prokaryotes for most of their life cycle (Costerton et al., 1995).

The main advantage of bacterial biofilms in relation to multicellularity of higher organisms lies in the relative higher freedom degree of the cells living in these communities. Contact and continuity are ensured because cells are embedded into a self-produced extracellular polymeric matrix (for differences in its composition, see Hobley et al., 2015) that acts as a connective tissue; however, differentiation is relatively limited. In higher organisms, cell differentiation is necessary for workflow partitioning. Actually, single cells belonging to multicellular organisms (a part from stem cells) cannot perform all the functions encoded in their genomes since most activities are repressed to address energy toward very specific tasks, according to the tissue/organ they belong, such as thyroid hormone synthesis, keratin production, and so on. They acquire a strong tissue identity and do not move from the site they live except during metastasis. The paradigm of single-species biofilm-living bacteria is different: (i) each cell does not deprive itself of the majority of its physiological functions, (ii) differentiation giving rise to a certain degree of phenotypic heterogeneity is generally a reversible event or only concerns some clones, and (iii) the process of living in community can be stopped, allowing some of the cells to revert back to the planktonic lifestyle (Jefferson, 2004).

It is worth highlighting that biofilm-living cells display a different gene expression profile from planktonic cells: as an example, a higher expression of genes involved in iron metabolism is observed since iron level is critical for expression of adhesion factors important in biofilm formation (Nakamura et al., 2016). A second feature that differs is motility: in *B. subtilis*, the operon involved in biofilm matrix biosynthesis also encoded an inhibitor of motility demonstrating that sessile lifestyle and planktonic lifestyle are oppositely regulated since matrix-embedded lifestyle hinders movements (Blair et al., 2008). However, transcriptomic data also confirm that flagella-related gene expression is dependent on the biofilm growth phase, highlighting the different importance of flagella during the adherence, maturation, and dispersal steps (Nakamura et al., 2016). Curiously, recently dispersed planktonic cells proved to be different both from biofilm parent cells and from truly planktonic ones (Guilhen et al., 2016, 2017), being more similar to cells giving rise to biofilm formation (Sauer et al., 2002).

It is evident from the above reported data that when talking about biofilm, considering the dynamic temporal evolution of phenotypes is of fundamental importance. The three main phases of a biofilm life, namely, adherence, maturation, and dispersal, are consistent with different phenotypes, similarly with what occurs in higher animals during aging (Steenackers et al., 2016). However, also at each developmental stage, bacterial subpopulations may arise, generating cell heterogeneity finalized to cooperation and to obtain best fitness for the whole community. Van Gestel et al. (2015) proposed the term “division of labor” to indicate the functions of cells specialized in different tasks and based on cell types displaying different gene expression profiles.

It is well recognized that differentiation into various cell phenotypes is mainly due to environmental gradients. In heterogeneous biofilms (made up of cells belonging to different species and, sometimes, different kingdoms), cells colonize different biofilm areas in relation to their oxygen demand, acidic tolerance, and metabolic features (Watnick and Kolter, 2000). Nevertheless, also in single-species biofilms, cell adaptation to chemical gradients can trigger differences in gene expression as well as mutation and selection for the fittest variant resulting in different spatial pattern formation among phenotypes (Stewart and Franklin, 2008). However, cells responding differently to the same spatial and temporal environmental conditions indicate that local physiological adaptation is not the only environmental factor affecting the cell differentiation at a metabolic/biosynthetic level.

Van Gestel et al. (2015) studied two bacterial models: *B. subtilis* and *P. aeruginosa*. In *P. aeruginosa* biofilm, two phenotypes of motile and non-motile cells concur to produce a mushroom-shaped structure. Non-motile cells, a subpopulation that is low metabolically active but has a high cell density, give rise to a sort of “stalk.” The stalk cells mainly produce QS signals, siderophores, exopolysaccharides (EPS), and surfactants (rhamnolipids) that become public goods available for the entire population. After a period of about 4 days, a second phenotype of motile cells appears and migrate, probably by twitching motility-mediated chemotaxis, on the top of stalk cells to have access to more nutrients, thus giving rise to the formation of “caps” in a mushroom-shaped three-dimensional biofilm. It has been observed that among non-motile stalk bacteria, sacrificial cells also appear (before the motile ones) and undergo cell lysis, thus liberating their DNA. This extracellular DNA is localized at the edge of the stalk and has the function, together with the surfactants, to facilitate climbing by the motile cells on the top of stalk cells. How this occurs is not fully understood; however, DNase treatment prevents climbing and exogenous DNA supply restores the migration (Van Gestel et al., 2015). The signal triggering cell lysis in sacrificial cells is a QS quinolone molecule produced by the stalk cells (Van Gestel et al., 2015). Besides low metabolically active, sacrificial, and motile cells, persister cells can be part of a biofilm as well. These are steady-state cells that survive without growing, setting their metabolism to minimal levels and that become resistant against environmental stressors (such as antibiotics), thus ensuring survival of the population in case of stress (Balaban et al., 2004). It is evident that these heterogeneous subpopulations contribute inside a biofilm to control regulatory circuits resulting in cooperative interactions finalized to work in concert and to improve fitness. However, about 7 days after the biofilm establishment, when waste catabolites begin to accumulate or when nutrients become scarce, some cells die whereas the most metabolically active ones start to disperse reverting to planktonic cells to look for a new nutrient-rich place to establish (Van Gestel et al., 2015). In this case, cell lysis occurs after induction (by QS signaling) of a filamentous prophage that is generally non-lytic in planktonic-style living cells (Webb et al., 2003). Cell lysis, together with extracellular matrix degradation, enhances the total number of dispersal units, while surfactants, such as rhamnolipids, produced by the stalk cells before dying, can facilitate cell dispersal. The subpopulation of dispersing cells remains viable, increasing the synthesis of flagellin and polysaccharide lyases (hydrolytic enzymes that break down the biofilm matrix), allowing dispersion from the matrix, and improving the possibility to move away and colonize new habitats (Boyd and Chakrabarty, 1994; Petrova and Sauer, 2016). This cooperative interaction should result in an emergent benefit supporting the migrating cells.

In the *B. subtilis* model, three phenotypes have been described: (i) matrix (EPS)-producing cells that also produce communication signals, proteases, and bacteria-directed toxins; (ii) motile cells; and (iii) sporulating cells. There is a sort of spatial distribution (although not so well-defined as in *P. aeruginosa*) where matrix producers stay in the center, motile cells on the edges and sporulating cells on the top of biofilms (Vlamakis et al., 2008). Here, again, when the biofilm grows old, a division of labor can be observed in which altruistic producers, sacrificial cells, and survivors (migrating but especially sporulating cells) are simultaneously present. Indeed, toxins secreted by the producers can kill sibling (unintentional sacrificial cells) that do not express the necessary immunity genes (Ellermeier et al., 2006) resulting in enhanced availability of nutrients that can delay sporulation. Both proteases and toxins can supply nutrients (degraded matrix and intracellular content of siblings) to accomplish sporulation and, in case, migration. Nevertheless, some sacrificial cells within *B. subtilis* biofilms also set up an autolytic behavior that is independent of the toxin and possibly comparable to programmed cell death: actually, caspase-like enzymes inducing apoptosis have also been described in prokaryotes (Rice and Bayles, 2003, 2008). The interaction between various subpopulations that occurs during biofilm growth promotes behaviors that prove to be cooperative, revealing population plasticity that finally results in enhanced survival and fitness of the overall community.

## Conclusion

By a continuous flow of mass and energy, as biogeochemical agents, as symbionts of plants and animals, as dead organic matter degraders, bacteria support essential functions that provide successful and sustainable ways of living on the Earth. Nevertheless, bacteria have to cope with an extremely changing environment (pH, temperature, and oxygen may rapidly change), facing a high number of challenges (nutrient scarcity, phage and/or host immune system attack, and environmentally released toxic xenobiotic compounds) and paying high costs to successfully overcome difficulties. According to some reports, competition and single-cell interest are prevalent during scarcity, whereas according to others, scarcity can favor bacterial cooperation and the formation of communities to ensure survival. However, it has to be underlined that cooperation, although sometimes helping in counteracting harsh conditions, is not free from conflicts.

Analyzing the strategies that bacteria use to overcome a variety of environmental stresses and to adjust reciprocal relationships can shed light on how evolutionary forces have shaped a winning model of life. Earning energy and resources, anticipating, cooperatively interacting with other bacterial cells to achieve systems of higher degree of complexity, sharing, and recycling seem to be the rule in prokaryote life. The main concept that emerges by observing bacteria is the necessity of a continuous adaptation to face new scenarios in a fast-changing environment. Each new difficulty encountered (phage attack, “cheaters,” and host immune system) can be an opportunity for evolution. The present environmental crisis (global warming, demographic pressure, desertification, and ocean acidification) also compels humans to face fast-changing conditions, seldom experimented before. The behaviors that evolution has selected in bacteria can suggest, among multiple possible strategies that humans can set up, interesting paths on how to overcome the present challenges.

## Author Contributions

The author confirms being the sole contributor of this work and has approved it for publication.

## Conflict of Interest

The authors declare that the research was conducted in the absence of any commercial or financial relationships that could be construed as a potential conflict of interest.
